# Stabilization of δ-like Bi_2_O_3_ Phase at Room Temperature in Binary and Ternary Bismuthate Glass Systems with Al_2_O_3_, SiO_2_, GeO_2_, and B_2_O_3_

**DOI:** 10.3390/ma17164023

**Published:** 2024-08-13

**Authors:** Viktoriia Vlasenko, Maciej Nowagiel, Marek Wasiucionek, Tomasz K. Pietrzak

**Affiliations:** Faculty of Physics, Warsaw University of Technology, Koszykowa 75, PL-00-662 Warsaw, Poland; viktoriia.vlasenko.stud@pw.edu.pl (V.V.);

**Keywords:** bismuth oxide, glass–ceramics, nanocrystallization, phase stabilization

## Abstract

Recently, it was shown that the nanocrystallization of $Bi_{2}O_{3}$ glasses with the addition of $SiO_{2}$ and $Al_{2}O_{3}$ leads to the stabilization of the δ-like $Bi_{2}O_{3}$ phase at least down to room temperature, which is significantly below its stability range in bulk form. In this research, we investigated the properties of bismuthate glasses synthesized with various glass-forming agents such as $SiO_{2}$, $GeO_{2}$, $B_{2}O_{3}$, and $Al_{2}O_{3}$. It was demonstrated that vitrification of all these systems is possible using a standard melt quenching route. Furthermore, we investigated the crystallization processes in pristine glasses upon increasing the temperature and the thermal stability of arising phases using thermal analysis and high-temperature XRD in situ experiments. It was shown that it is possible to stabilize crystallites’ isostructures with δ-$Bi_{2}O_{3}$ embedded in a residual glassy matrix down to room temperature. The temperature range of the appearance of the δ-like phase strongly depended on the nominal composition of the glasses. We postulate that the confinement effect depends on the local properties of the residual glassy matrix and its ability to introduce sufficient force to stretch the structure of the δ-like $Bi_{2}O_{3}$ phase in the nanocrystallites.

## 1. Introduction

Ionic conductors are a class of materials where positively or negatively charged ions play the role of charge carriers. They have drawn attention not only due to interesting physical and chemical phenomena but also due to various applications, e.g., Li^+^ or Na^+^ conductors may serve as electrodes or electrolytes for lithium [1] and sodium [2] ion batteries. Recently, Mg^2+^ [3] or K^+^ [4] conductors have been studied as materials for the new generation of magnesium or potassium batteries. Oxygen conductors find applications in solid oxide fuel cells (SOFCs) or gas sensors [5]. Ag^+^ conductors can be applied, e.g., as chemical sensors [6].

Usually, high ionic conduction is a desirable property of the aforementioned materials. Unfortunately, some highly conducting phases are stable only at elevated temperatures. ${\mathrm{Bi}}_{2}O_{3}$ exists in four primary polymorphs: α (monoclinic), β (tetragonal), γ (body-centered cubic), and δ (face-centered cubic) [7,8]. These polymorphs possess distinct physical characteristics, including electrical, optical, and photoelectrical properties. The α phase is stable in a wide temperature range (including room temperature) but exhibits modest oxygen ion conductivity. At temperatures above 729 °C, ${\mathrm{Bi}}_{2}O_{3}$ exhibits transition into the δ phase and subsequently melts at 824 °C. The δ $Bi_{2}O_{3}$ phase exhibits a face-centered cubic (fcc) fluorite structure, where 25% of the oxygen positions remain unoccupied [9]. This results in a relatively high mobility of oxygen ions. Furthermore, the electronic configuration of ${\mathrm{Bi}}^{3+}$ includes a loose pair of 6s electrons, which results in a highly polarizable cation lattice. That is why the δ $Bi_{2}O_{3}$ phase is particularly noted for its exceptionally high oxygen ionic conductivity—ca. 1 S/cm within its narrow stability range [10].

Therefore, much effort has been put into stabilizing the δ $Bi_{2}O_{3}$ phase to lower temperatures. Doping or preparing solid solutions with trivalent rare earth elements (e.g., Y, Tm, Er, Ho, Yb, and Dy) seems to be the most popular approach so far [10,11,12,13,14]. Alternatively, the δ $Bi_{2}O_{3}$ phase can be obtained at room temperature in the form of a thin film [15,16,17,18]. Therefore, a large number of the δ $Bi_{2}O_{3}$ phase reference patterns is available in diffraction databases, with the lattice parameter *a* spanning from 5.21 Å (ICDD card no. 04-028-0882) through to 5.26 Å (ICDD card no. 03-065-3319), as well as 5.45 Å (ICDD card no. 01-076-2478) to 5.66 Å (ICDD card no. 04-005-4788). As bismuth atoms have a much larger atomic number than oxygen (83 vs. 16), the X-ray diffraction patterns are dominated by the positions of the bismuth, and very little can be said about the oxygen sublattice. In particular, the high mobility in the oxygen network may not be maintained, even if the XRD pattern agrees with the reference one. Therefore, in a situation when there is no evidence of high oxygen conduction, the term ‘δ-*like* $Bi_{2}O_{3}$ phase’ should be used. So, we will do so in this paper.

In recent decades, several groups have reported the possibility of extending downwards the stability range of certain high-temperature crystalline phases of simple compounds using novel approaches. The first research project by Tatsumisago et al. [19] showed that rapid cooling (exceeding $10^{5}$ K/s) of silver borate glasses rich in AgI resulted in the formation of glass–ceramic composites. These composites contained nanosized crystallites of α AgI evenly dispersed within a glassy matrix. Remarkably, the α phase, typically stable only above 147 °C in a pure form, was maintained at room temperatures.

Interestingly, the effect of stabilizing the δ-like $Bi_{2}O_{3}$ phase was observed not only in polycrystalline solids but also in bismuthate glasses. The annealing of ${\mathrm{Bi}}_{2}O_{3}$ glasses slightly above their glass transition temperature leads to the formation of nanosized crystallites of the δ-like ${\mathrm{Bi}}_{2}O_{3}$ phase, which are confined within the glassy matrix [20]. The resultant materials remained stable at room temperature for extended periods, even exceeding a year [21]. It was also demonstrated that the appearance of the $Bi_{2}O_{3}$ phases (namely, β, γ, and δ) depended on the heat treatment protocol and the content of $SiO_{2}$ additives. It was speculated that Si and Al additives do not incorporate into δ-like $Bi_{2}O_{3}$ nanograins but remain in the residual glassy matrix. Rather, it is determined by the size and strain effects in the nanograins embedded in the glassy matrix.

## 2. Bismuthate Glasses and Nanomaterials

Bismuthate glasses gain considerable interest, because $Bi_{2}O_{3}$ is recognized as one of the most important constituents for the design and control of the thermal and optical properties of oxide glasses. They are studied as innovative materials for low-temperature melting sealing applications and photonic devices (e.g., [22,23] and the references therein). Glass systems similar to those reported in this paper have also been studied by other groups. Glassy samples containing 99.5% [$x{\mathrm{GeO}}_{2}\cdot \left(1-x\right)$ ${\mathrm{Bi}}_{2}O_{3}$] with a 0.5% addition of MnO were synthesized using the melt quenching method (*x* ranged from 0.20 to 0.875) [24]. System $x{\mathrm{Bi}}_{2}O_{3}\cdot \left(100-x\right){\mathrm{GeO}}_{2}$ (in mol. %, *x* = 10, 15, 20, and 25) was studied regarding the optical properties and the structure of glass, as well as the role of Bi in network formation [25]. Bismuth alumino-borate glasses have been shown to be feasible to obtain in a broad compositional range [26]. Bismuth silicate materials have been studied, e.g., by Gattow. Rapid cooling of the melt, however, led to instant crystallization of δ-like $Bi_{2}O_{3}$ rather than glass formation [27]. Sanz studied i.a. bismuth silicate materials of composition 6$Bi_{2}O_{3}$–$SiO_{2}$ and 2$Bi_{2}O_{3}$–3$SiO_{2}$. Various synthesis conditions were investigated—the temperature of melting was changed in a wide range, and a few crucible types were used. In most of the cases, glassy material was produced [28]. Todea and Simon reported a study on bismuth silicate glasses and glass–ceramics obtained in the annealing process. The $SiO_{2}-Bi_{2}O_{3}$ system in a large composition range from 18:1 to 6:7 Bi:Si atomic ratio was investigated. Interestingly, in samples containing 30–60 mol% $SiO_{2}$, crystallization of the δ-like $Bi_{2}O_{3}$ phase was observed [29,30,31]. The glass network structure and optical parameters of bismuth silicate glasses have also been studied by Ahlawat et al. [32]. From these works, it was concluded that Bi is incorporated into the glass network structure to form [$BiO_{6}$] octahedra or [$BiO_{3}$] pyramids depending on the $SiO_{2}$ content.

The compositions of bismuthate glasses studied in the literature varied noticeably, and the authors usually did not focus on the crystallization processes occurring in these glasses. Therefore, we decided to investigate whether other good glass formers added to $Bi_{2}O_{3}$ may result in crystallization of the δ-like $Bi_{2}O_{3}$ phase upon annealing. In this work, we synthesized several binary and ternary bismuthate glass systems with different glass-forming dioxides and trioxides. The syntheses were followed by a systematic study of those systems that focused on the crystallization processes occurring during annealing. The initial goal was to synthesize the samples in the glassy form, with a significant content of $Bi_{2}O_{3}$. As a preliminary study, we investigated several other potential systems, e.g., $Bi_{2}O_{3}-GeO_{2}$, but we have not obtained initially glassy samples. For clarity, those results are not reported in this paper. Here, the investigation of the following systems is reported: $Bi_{2}O_{3}-Al_{2}O_{3}-GeO_{2}$, $Bi_{2}O_{3}-Al_{2}O_{3}-SiO_{2}$, $Bi_{2}O_{3}-B_{2}O_{3}-SiO_{2}$, and $Bi_{2}O_{3}-SiO_{2}$. We show their glass-forming properties and thermal stability, as evidenced by differential thermal analysis, and we will observe the appearance of crystalline phases upon proper thermal treatment. Eventually, we aim to better understand the factors originating from the glass network that influence the stabilization mechanism of the confined δ-like $Bi_{2}O_{3}$ phase.

## 3. Materials and Methods

All bismuthate materials studied in this paper were synthesized using a typical melt quenching method. A fixed atomic ratio between Bi and additive atoms was maintained in nominal compositions. In binary system, the ratio was 4:3. In ternary systems, namely, $Bi_{2}O_{3}-A^{\prime }O_{2}-A^{\prime \prime }2O_{3}$, the 20:8:7 ratio between B, $A^{\prime }$, and $A^{\prime \prime }$ was kept (which means Bi to all additive atoms ratio of 4:3, as well). According to our previous works [20,21], this is the ratio at which the δ-like phase is most likely to crystallize from the glassy state. Lower contents of glass formers would result in poor vitrification of the samples.

The stoichiometric amounts of the reagents (bismuth(III) oxide—Acros Organics (Geel, Belgium), >99.9%; silicon dioxide—Supelco (Steinheim, Germany), >99.7%; aluminum oxide—Acros Organics, >99%; germanium dioxide—Angene (London, UK), >99.99%, boric acid—Roth (Karlsruhe, Germany), >99.5%) were carefully homogenized in a mortar with a pestle. The batches were put into platinum crucibles and melted at 1100–1350 °C for 15 min. The compositions, the labels used in this work, and the temperatures of syntheses are listed in Table 1.

The amorphousness of as-synthesized samples was verified with X-ray diffractometry (XRD). Powdered samples were measured in Bragg–Brentano geometry in Panalytical Empyrean apparatus using CuK_α_ radiation in the 2θ range from 5 to 115° (Malvern, UK). Subsequently, the crystallization processes were observed in situ using a Philips X’Pert Pro diffractometer equipped (Cambridge, MA, USA) with an Anton Paar HTK-1200 high-temperature furnace (Ashland, VA, USA). The measurements were conducted in the air. Acquisition of a single pattern lasted ca. 30 min and was performed after 20 min of the temperature stabilization. At first, the temperature step was 10 °C, and the maximum temperature was set to 750 °C. To obtain an optimized phase composition (preferably pure δ $Bi_{2}O_{3}$), the thermal treatment protocols were subsequently prepared individually for different samples according to their DTA traces, as described in the next paragraph.

The temperatures of glass transition and crystallization processes in powdered samples were investigated using differential thermal analysis (DTA). TA Instruments SDT Q600 (New Castle, DE, USA) apparatus was used. The measurements were carried out in a synthetic air flow with a heating rate of 10 °C/min.

## 4. Results

### 4.1. X-ray Diffractometry—Pristine Glasses

**Figure 1:**
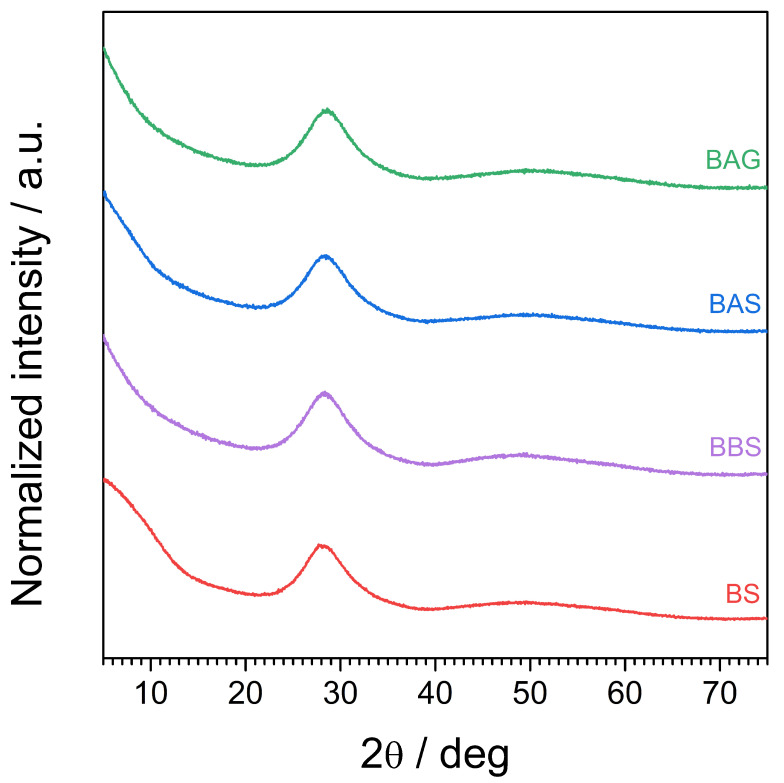
XRD patterns of as-synthesized samples.

The XRD patterns of the as-synthesized samples are shown in Figure 1. All the diffractograms were typical for glassy materials and proved the successful syntheses of all the studied glasses. No diffraction lines were visible, but only broad halos centered at ca. 28°. Additionally, one can conclude that $SiO_{2}$ is not essential to obtain a bismuthate glass. In particular, a glassy sample of ternary $Bi_{2}O_{3}-Al_{2}O_{3}-GeO_{2}$ system could be synthesized as well.

### 4.2. Differential Thermal Analysis (DTA)

**Figure 2:**
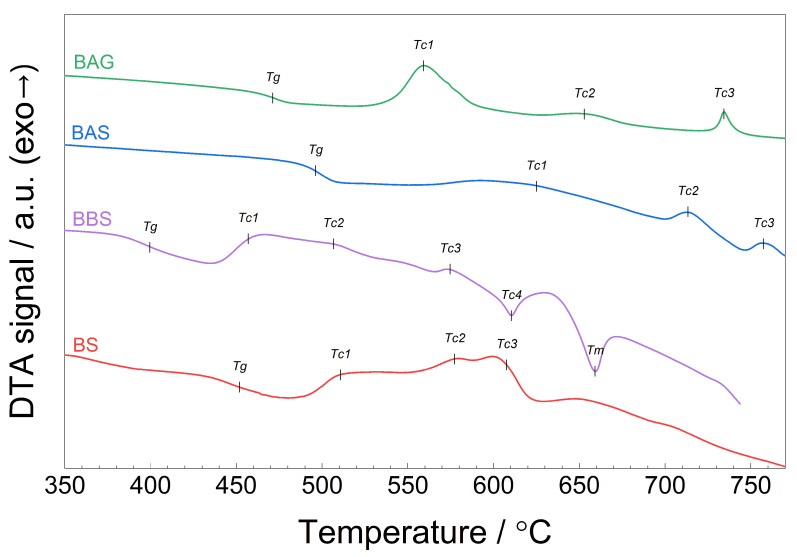
DTA traces of as-synthesized glasses. The measurements were carried out in the air with the heating rate of 10 °C/min.

DTA traces of the as-synthesized samples are shown in Figure 2. The DTA curves are characteristic for glassy materials, which is in agreement with their XRD patterns. One can see a step-like endothermic shift of the curves, which indicates the glass transition. The glass transition was followed by an exothermic peak corresponding to the first crystallization process in the glass. Subsequently, further exothermic or endothermic crystallization processes appeared. The glass transition temperatures ($T_{g}$) and temperatures of the extrema of the crystallization peaks ($T_{c}$) for all studied glasses are shown in Table 2. Within the measurements range (namely, up to 770 °C), we observed the melting process only in BBS glass. The other samples remained solid during the whole experiment. The assignment of the crystallization processes was not always obvious when looking at the DTA traces only. Therefore, the analysis was made in reference to high-temperature XRD analyses. Typically, crystallization is an exothermic process, especially in glass, when atoms move to reach a local net potential minimum. However, phase transitions between different crystalline phases can be endothermic, as, e.g., in α→δ $Bi_{2}O_{3}$ [33].

One can see that the characteristic temperatures varied with the compositions of the glasses. Comparing the BAG and BAS batches, we see that substituting germanium oxide for silicon oxide lowered the characteristic temperatures (glass transition and crystallization). A similar effect is observable when aluminum oxide was replaced by boron oxide (BAS vs. BBS). One can also notice that the characteristic temperatures of the binary composition BS were in between the BBS (lower temperatures) and BAS (higher temperatures). These observations are intuitive, because $B_{2}O_{3}$ is a very good glass former, whereas $Al_{2}O_{3}$ can be considered as an intermediate. The glass transition temperature depends on the topological properties of a random glass network. Thus, borate glasses are known for having a relatively low glass transition temperature [34], whereas the addition of $Al_{2}O_{3}$ may lead to a significant increase in the glass transition temperature (as reported, e.g., in Ref. [35]).

### 4.3. High-Temperature X-ray Diffractometry

**Figure 3:**
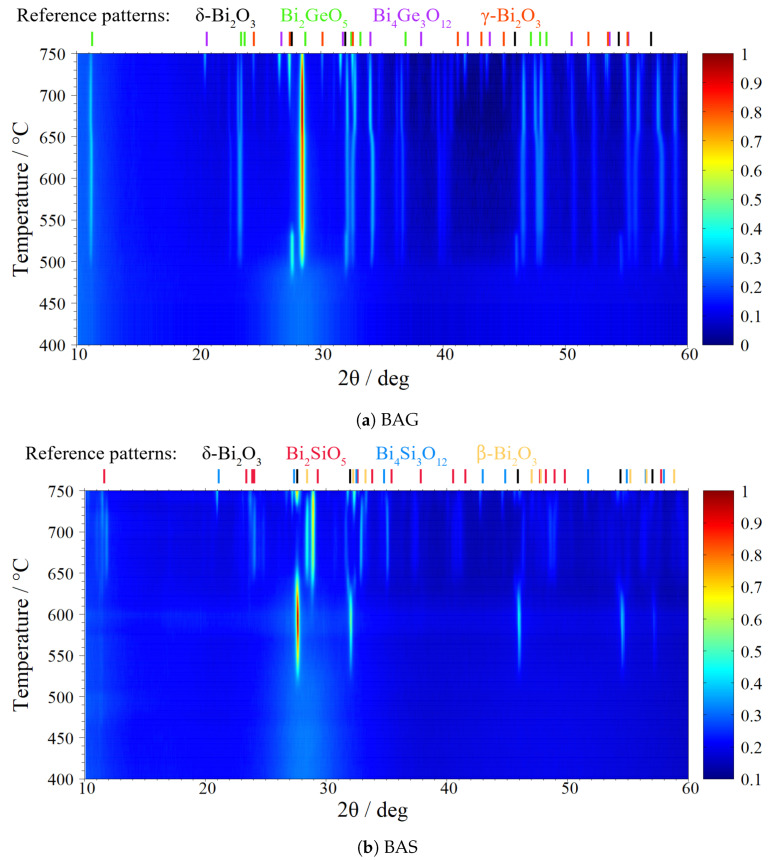

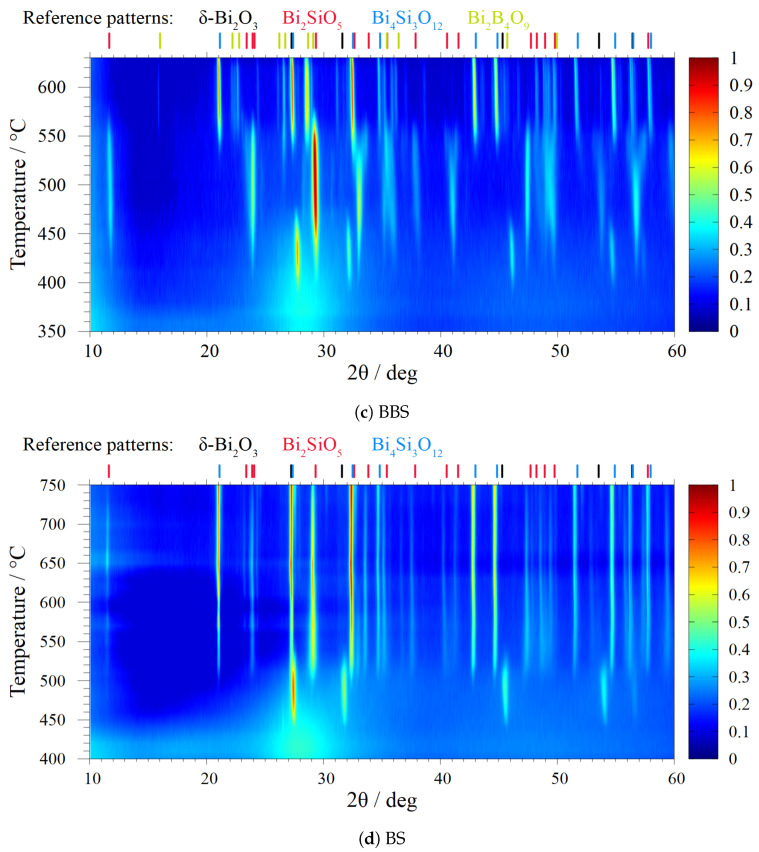
XRD patterns collected in situ upon heating the pristine glasses (BAG—(**a**); BAS—(**b**); BBS—(**c**); BS—(**d**)) to 750 °C. The positions of main lines ascribed to crystalline phases identified in the samples are shown at the top of each plot. Please refer to the text for their descriptions. The color scale indicates the normalized square root of the intensity.

To identify the crystallization processes visible in the DTA traces (Figure 2), the diffraction patterns of the samples were collected in situ upon their heating from room temperature to 750 °C—with an exception of BBS, whose investigation was limited to 630 °C due to its lower melting point). The results are presented in Figure 3a–d. For clarity, phases identified in specific temperature ranges are summmarized in Table 3. One should be aware of the fact that the HT-XRD measurements were carried out at equilibrium conditions, whereas the DTA measurements were conducted with a fixed heating rate of 10 °C/min. Therefore, it is natural that the temperatures observed in the nearly isothermal XRD experiment (the beginning of crystallization processes) were lower than the corresponding $T_{c}$ temperatures (maxima of crystallization peaks) determined from DTA.

The sample BAG (Figure 3a) remained glassy up to 470 °C. Subsequently, the δ-like $Bi_{2}O_{3}$ phase appeared. However, the stability range of this phase was very narrow. The intensities were largest around 490 °C and disappeared below 550 °C. At the same time, an orthorhombic $Bi_{2}GeO_{5}$ (ICDD card no. 00-039-0003) emerged. Thus, $T_{c1}$ can be attributed to the crystallization of these phases from the glassy matrix. Afterward, the intensity of the orthorhombic phase peaks increased, which can be attributed to the prolonged growth of crystal grains. This exothermic process is visible as a broad DTA peak with a maximum at $T_{c2}$. Around 650 °C, we can observe splitting of the peak at position ca. 23.3° into two peaks: at 23.20° corresponding to (310) plain and at 23.46° corresponding to ($\bar{1}$11) plain. The same happened to the peak at ca. 47.7°; it split into two lines: at position 47.50°corresponding to (620) plain and at 47.98°corresponding to ($\bar{6}02$) plain. We assume that in this process, the lattice parameters of the orthorhombic $Bi_{2}GeO_{5}$ structure change unequally, causing the splitting of the aforementioned peaks. An analogous explanation was given by Bermeshev et al. for a similar observation of the crystalline samples from the same family at room temperature [36]. In our case, this splitting was temperature-induced and can be attributed to the $T_{c3}$ temperature. Above 720 °C, two new phases appeared: cubic γ $Bi_{2}O_{3}$ (ICDD card no. 00-045-1344) and cubic $Bi_{4}Ge_{3}O_{12}$ (ICDD card no. 04-012-7982). Therefore, the sample is a multiphase material with three crystalline phases present at 750 °C.

The sample BAS (Figure 3b) remained glassy up to ca. 510 °C, when the first diffraction lines ascribed to the δ-like $Bi_{2}O_{3}$ appeared. Their intensities systematically increased upon heating. This prolonged growth of the crystalline phase from the glassy matrix can be attributed to the $T_{c1}$ broad DTA peak. At 640 °C, two other phases appeared. At the same time, the Bragg lines of the δ-like phase decreased in intensity, and the share of the δ-like phase became negligible at ca. 680 °C. This transition corresponds to $T_{c2}$. Between 680 and 740 °C, we observed peaks at position 31.65° that could not be identified with satisfactory certainty. At 710 °C, lines corresponding to a cubic $Bi_{4}Si_{3}O_{12}$ (ICDD card no. 04-008-3527) began to appear, which are probably visible as $T_{c3}$ in the DTA curve. Above 740 °C, β $Bi_{2}O_{3}$ disappeared, and simultaneously, we observed the reappearance of the δ-like phase. The reappearance is not a surprise, as that is the thermodynamical stability region of bulk δ $Bi_{2}O_{3}$. This phenomenon is probably beyond the temperature range of the DTA curve; thus, we did not observe an endothermic peak associated with this transition. Apart from the δ-like phase, traces of γ $Bi_{2}O_{3}$ were also visible. The behavior of this glass differs from the crystallization process occurring in the $Bi_{2}O_{3}-SiO_{2}-Al_{2}O_{3}$ glass reported in Ref. [20], where the δ-like $Bi_{2}O_{3}$ also disappeared at ca. 630–650 °C, but it transitioned into β $Bi_{2}O_{3}$ only. The aforementioned samples differed in their synthesis. In this work, the sample was melted in a platinum crucible, and $SiO_{2}$ and $Al_{2}O_{3}$ additives were added in controlled amounts. In the referred work, the batch was melted in porcelain crucibles from which the additives originated.

The sample BBS (Figure 3c) remained glassy up to 380 °C. Subsequently, at 390 °C, the δ-like $Bi_{2}O_{3}$ phase appeared, which was almost immediately followed by $Bi_{2}SiO_{5}$ (ICDD card no. 04-019-9381). The δ-like $Bi_{2}O_{3}$ phase persisted until 470 °C, and $Bi_{2}SiO_{5}$ persisted until 570 °C. The crystallization and growth of these phases can be attributed to the DTA peak $T_{c1}$. Around 440 °C, peaks originating from $Bi_{2}SiO_{5}$ grew, while δ-like $Bi_{2}O_{3}$ vanished. This transition can be ascribed to $T_{c2}$. At 500 °C, lines that can be ascribed to $Bi_{2}B_{4}O_{9}$ (ICSD card no. 98-025-0427) appeared. This phase crystallized from the residual glass, so the process would have been exothermic ($T_{c3}$ peak). At 540 °C, lines that can be ascribed to $Bi_{4}Si_{3}O_{12}$ (ICDD card no. 04-008-3527) showed up. Simultaneously, the $Bi_{2}SiO_{5}$ phase disappeared and probably transformed into $Bi_{4}Si_{3}O_{12}$. This phenomenon may be tentatively interpreted as $T_{c4}$ in the DTA curve. The measurement ended at 630 °C, because the sample would have started to melt at higher temperatures.

The sample BS (Figure 3d) remained glassy up to 440 °C. At 460 °C, diffraction lines ascribed to the crystallization of δ-like $Bi_{2}O_{3}$ appeared ($T_{c1}$). At this temperature, a broad amorphous halo was still visible in the pattern. Subsequent heating led to the appearance of an orthorhombic $Bi_{2}SiO_{5}$ (ICDD card no. 04-019-9381) and a cubic $Bi_{4}Si_{3}O_{12}$ (ICDD card no. 04-008-3527) phase at around 480 and 500 °C, accordingly. These transitions correspond well with the $T_{c2}$ and $T_{c3}$ DTA peaks.

In Figure 3a–d, we show the phase transitions observed upon heating the glasses to (in most cases) 750 °C. In all cases, no changes in the phase compositions were observed upon subsequently cooling the studied samples down to room temperature. In other words, the crystallized phases determined at the end of this HT-XRD in situ experiment also remained stable later at room temperature. This observation was fundamental for the optimization experiments described in the next section. Furthermore, from our previous experience with nanocrystallized glasses and experiments conducted for these materials, subsequent heating to the same or lower temperature does not lead to noticeable phase changes. In other words, the obtained phases remain stable in the temperature range below the heat treatment temperature from the first cycle.

### 4.4. Optimization of Crystallization Process

Having investigated the crystallization processes in all samples in a wide temperature range from room temperature to 750 °C, we attempted to propose optimized heat treatment protocols to our glassy samples in order to obtain a δ-like $Bi_{2}O_{3}$ phase presumably stabilized down to room temperature. The optimization was driven by the previous HT-XRD studies. However, several attempts were made at different conditions (i.e., the time and the temperature of heat treatment) to obtain satisfactory results—high phase purity, good crystallinity, or at least as high a δ-like $Bi_{2}O_{3}$ phase share as possible. Here, for clarity, we present only the final results. We started with an arbitrary time of 24 h to ensure the crystallization of all samples even at temperatures close to $T_{g}$. In the case of the BAG sample, annealing at 480 °C resulted in fast growth of the $Bi_{2}GeO_{5}$ secondary phase. At 460 °C, we did not observe any crystallization at all. Thus, 470 °C was a compromise between the growth of δ-like $Bi_{2}O_{3}$ and the secondary phase. Whereas a time shorter than 24 h would result in higher δ-like $Bi_{2}O_{3}$ phase relative share, it would also lead to lower crystallinity of the sample. In the case of the BAS sample, one hour of annealing was enough for good crystallinity. A longer time (24 h) and higher temperature (600 °C) resulted in undesired significant growth of the secondary phase. The BBS was annealed for 24 h at 390 °C, as it was the first temperature at which any crystalline peaks could be observed. After 24 h, the share of crystallinity reached a plateau. Thus, the results were satisfactory, and no further optimization was conducted. Annealing the BS sample for 3 h was enough to provide good crystallinity, and the only observed crystalline phase was δ-like $Bi_{2}O_{3}$. Annealing for longer times did not result in any change in the peaks’ intensity. Moreover, small traces of the secondary phase were observed at longer times.

**Figure 4:**
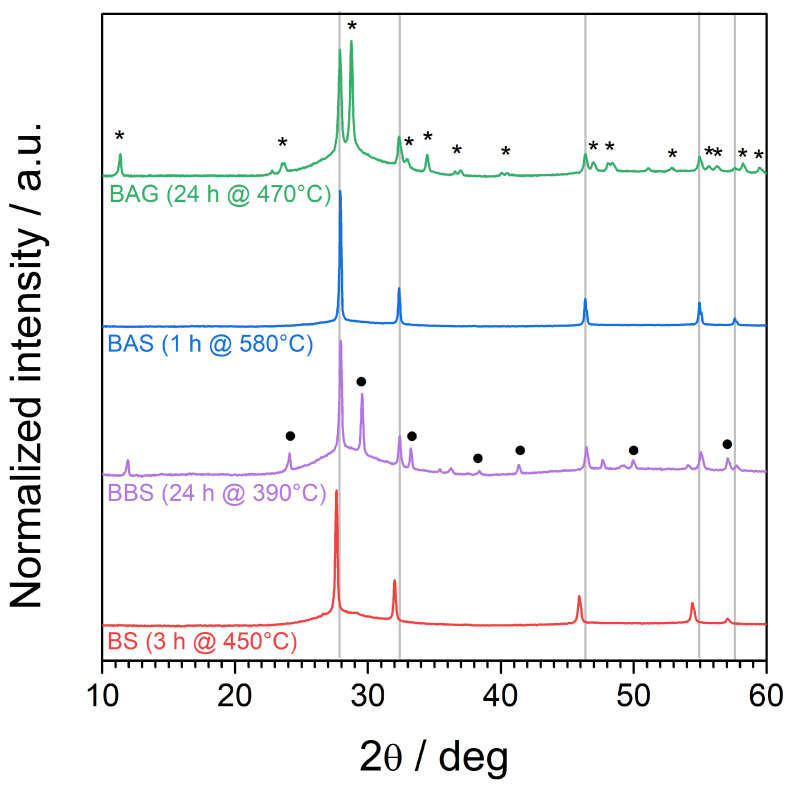
XRD patterns acquired at room temperatures of glassy samples after optimized heat treatment: BAG (24 h at 470 °C), BAS (1 h at 580 °C), BBS (24 h at 390 °C), and BS (24 h at 450 °C). Vertical gray lines indicate the position of the diffraction lines of δ $Bi_{2}O_{3}$ with the lattice parameter a=5.53 Å (ICDD card no. 00-052-1007). Asterisks denote diffraction lines ascribed to $Bi_{2}GeO_{5}$ (ICDD card no. 00-039-0003), and full circles correspond to the diffraction lines of $Bi_{2}SiO_{5}$ (ICDD card no. 04-019-9381).

In Figure 4, we present diffractograms of the glass samples after the optimized heat treatment protocol, which were acquired at room temperature. The δ-like $Bi_{2}O_{3}$ phase with high purity was obtained in BS glass after annealing for 3 h at 450 °C. The lattice parameter was a=5.60 Å (at room temperature). The pure δ-like $Bi_{2}O_{3}$ was also obtained in the sample BAS after annealing at 580 °C for 1 h. The lattice parameter was equal to a=5.53 Å (at room temperature). Furthermore, a signal originating from the residual glassy matrix was still observed in the form of an amorphous halo at low angles beneath the crystalline peaks. In the sample BBS after 24 h of annealing at 390 °C, we still observed a significant amorphous halo and reflexes originating from δ-like $Bi_{2}O_{3}$ (a=5.53 Å at room temperature) and $Bi_{2}SiO_{5}$. Several minor diffraction lines not ascribed to the aforementioned phases could not be reliably ascribed to any other bismuth and/or silicon-containing phases. As we did not observe any phases related to $B_{2}O_{3}$; we may assume that boron oxide remains in residual glass rather than becoming incorporated into grown crystalline phases. Likewise, we did not manage to obtain the BAG sample with the pure δ-like $Bi_{2}O_{3}$ phase. The sample kept at 470 °C for 24 h contained a comparable share of $Bi_{2}GeO_{5}$. The lattice parameter of the δ-like $Bi_{2}O_{3}$ phase was equal to a=5.53 Å (at room temperature).

The average size of the grains of the δ-like $Bi_{2}O_{3}$ phase was estimated in each aforementioned sample using the Scherrer equation [37]. The values are presented in Table 4. In all cases, the grain sizes were considerably below 100 nm. We did not see an obvious correlation with the heating time or annealing temperature. Usually, one would expect the appearance of large grains in samples annealed for a long time at higher temperatures.

## 5. Discussion and Conclusions

In addition to previously studied ternary $Bi_{2}O_{3}-SiO_{2}-Al_{2}O_{3}$ (BAS) bismuthate glasses [20,21], in this work, we have shown that it is possible to obtain other bismuthate glasses in a binary $Bi_{2}O_{3}-SiO_{2}$ (BS) system, as well as in $Bi_{2}O_{3}-B_{2}O_{3}-SiO_{2}$ (BBS) or $Bi_{2}O_{3}-Al_{2}O_{3}-GeO_{2}$ (BAG) ternary systems in which δ-like $Bi_{2}O_{3}$ phase crystallization is observed during annealing. Good glass formers, namely, $SiO_{2}$, $B_{2}O_{3}$, or $GeO_{2}$, play an essential role in the vitrification of the materials upon quenching. The glass formers used have a significant influence on the glass transition and crystallization temperatures investigated in the differential thermal analysis experiments.

The crystallization processes upon heating the glasses were carefully investigated. Each system showed different behavior. However, the common feature of all the studied compositions was the appearance of the δ-like $Bi_{2}O_{3}$ phase from the glassy state at different temperatures and with different thermal stabilities. Remarkably, the lattice parameter of the δ-like $Bi_{2}O_{3}$ phase (at room temperature) in the BS sample was larger than in the other samples, namely, a=5.60 Å vs. 5.53 Å. In contrast, the BAS sample showed a tendency to stabilize larger grains in size, but the lattice parameter remained relatively small, i.e., 5.53 Å. We assume that this effect stems from a change in the thermodynamical stability conditions arising from the stretch induced by glassy matrices of different compositions and from the influence of the grain surfaces. The sample with the δ-like phase with a larger lattice parameter (namely, a=5.60 Å) was the only binary system studied in this work. We may speculate that the differences in the lattice parameter in ternary systems may be caused by the additional glass former, i.e., $B_{2}O_{3}$ or $Al_{2}O_{3}$. The exact mechanism is not clear at this moment, but it will be carefully investigated in future works.

Interestingly, the δ-like $Bi_{2}O_{3}$ phase appeared first during the heating process in all the studied samples and was followed or accompanied by $Bi_{2}SiO_{5}$ and $Bi_{2}GeO_{5}$ phases. These phases contain a significant amount of Si/Ge additive in theoretical structure. Even more Si/Ge/B additive content is characteristic for phases that emerged at the highest temperature ranges—$Bi_{4}Si_{3}O_{12}$, $Bi_{4}Ge_{3}O_{12}$, and $Bi_{2}B_{4}O_{9}$. The explanation of this behavior can be as follows. With increasing amounts of crystallites upon heating the samples, the amount of the residual glassy matrix becomes lower and lower. Thus, the processes that may occur are either the crystallization of new Si/Ge/B-rich phases or the incorporation of these glass-forming additives into the crystal structure of existing grains leading to their phase transition. Further in-depth TEM/EDS measurements would be required to carefully investigate this phenomenon.

In comparison to our previous works on ternary $Bi_{2}O_{3}-Al_{2}O_{3}-SiO_{2}$ bismuthate glasses [20,21], in this work, we studied the δ-like $Bi_{2}O_{3}$ phase confinement effect in other binary and ternary glass systems containing, among others, $GeO_{2}$ and $B_{2}O_{3}$. It was demonstrated that the δ-like $Bi_{2}O_{3}$ phase can be stabilized down to room temperature without the presence of $Al_{2}O_{3}$ (e.g., in BS and BBS systems) or $SiO_{2}$ (BAG system). While the nanocrystallites of the δ-like $Bi_{2}O_{3}$ phase were observed—at room temperature—in all studied systems in this work, the content of the aforementioned glass formers had a significant influence on the temperature of the δ-like $Bi_{2}O_{3}$ phase formation and the appearance of additional phases.

To conclude, we sustain our point of view (presented earlier in Refs. [20,21]) that the additives do not enter the structure of the δ-like $Bi_{2}O_{3}$ phase, as Al, B, Si, and Ge ions are considerably smaller than typical dopants used for the stabilization of this phase. At higher temperatures, when the δ-like $Bi_{2}O_{3}$ phase usually disappeared, we observed several bismuthate phases containing the additive elements, e.g., $Bi_{2}GeO_{5}$, $Bi_{2}SiO_{5}$, $Bi_{4}Si_{3}O_{12}$, $Bi_{4}Ge_{3}O_{12}$, and $Bi_{2}B_{4}O_{9}$. Rather, aluminum, boron, silicon, or germanium oxides are essential to form bismuthate glass and subsequently lead to the formation of $Bi_{2}O_{3}$ nanocrystallites embedded in a residual glassy matrix.

At this point, we find it difficult to quantitatively explain the influence of the glassy matrix on the stabilization of the δ-like $Bi_{2}O_{3}$ phase confined as nanocrystallites in the studied samples. This work, however, has shown that this influence is significant. Therefore, qualitatively, we may argue that the confinement effect depends on the local properties of the residual glassy matrix and its ability to introduce sufficient force to stretch the structure of δ-like $Bi_{2}O_{3}$ in the nanocrystallites. Therefore, this phenomenon is worth further in-depth investigation.

## Figures and Tables

**Table 1:** Sample labels (IDs), their nominal compositions, and synthesis temperatures.

Sample ID	Nominal Composition	Synthesis Temperature
BAG	$10Bi_{2}O_{3}\cdot 3.5Al_{2}O_{3}\cdot 8GeO_{2}$	1350 °C
BAS	$10Bi_{2}O_{3}\cdot 3.5Al_{2}O_{3}\cdot 8SiO_{2}$	1100 °C
BBS	$10Bi_{2}O_{3}\cdot 3.5B_{2}O_{3}\cdot 8SiO_{2}$	1100 °C
BS	$10Bi_{2}O_{3}\cdot 15SiO_{2}$	1200 °C

**Table 2:** Characteristic temperatures of glass transition ($T_{g}$) and crystallization processes ($T_{c}$) determined from DTA traces of all studied glasses. All values are given in degrees Celsius (°C). The heating rate was 10 °C/min.

Sample ID	$T_{g}$	$T_{c1}$	$T_{c2}$	$T_{c3}$	$T_{c4}$
BAG	471	560	658	735	-
BAS	499	625	718	760	-
BBS	400	457	508	576	610
BS	450	508	578	608	-

**Table 3:** Identified phases during heat treatment of the studied glasses. For reference patterns, see Figure 3. UP denotes unidentified phase—no matching reference pattern was found.

Sample	Temperature Range/°C	Identified Phase
BAG	<470	glass
	470–490	δ $Bi_{2}O_{3}$
	490–550	δ $Bi_{2}O_{3}$, $Bi_{2}GeO_{5}$
	550–720	$Bi_{2}GeO_{5}$
	720–750	γ $Bi_{2}O_{3}$, $Bi_{4}Ge_{3}O_{12}$, $Bi_{2}GeO_{5}$
BAS	<510	glass
	510–640	δ $Bi_{2}O_{3}$
	640–680	δ $Bi_{2}O_{3}$, β $Bi_{2}O_{3}$, $Bi_{2}SiO_{5}$
	680–710	β $Bi_{2}O_{3}$, $Bi_{2}SiO_{5}$, UP
	710–740	β $Bi_{2}O_{3}$, $Bi_{2}SiO_{5}$, $Bi_{4}Si_{3}O_{12}$, UP
	740–750	δ $Bi_{2}O_{3}$, $Bi_{2}SiO_{5}$, $Bi_{4}Si_{3}O_{12}$, γ $Bi_{2}O_{3}$
BBS	<390	glass
	390–470	δ $Bi_{2}O_{3}$, $Bi_{2}SiO_{5}$
	470–500	$Bi_{2}SiO_{5}$
	500–540	$Bi_{2}SiO_{5}$, $Bi_{2}B_{4}O_{9}$
	540–570	$Bi_{2}SiO_{5}$, $Bi_{2}B_{4}O_{9}$, $Bi_{4}Si_{3}O_{12}$
	570–630	$Bi_{2}B_{4}O_{9}$, $Bi_{4}Si_{3}O_{12}$
BS	<440	glass
	440–480	δ $Bi_{2}O_{3}$
	480–500	δ $Bi_{2}O_{3}$, $Bi_{2}SiO_{5}$
	500–540	δ $Bi_{2}O_{3}$, $Bi_{2}SiO_{5}$, $Bi_{4}Si_{3}O_{12}$
	540–750	$Bi_{2}SiO_{5}$, $Bi_{4}Si_{3}O_{12}$

**Table 4:** Average grain sizes of δ-like $Bi_{2}O_{3}$ grains in crystallized glasses estimated from XRD patterns using Scherrer method.

Sample	Annealing Protocol	Average Grain Size/nm
BAG	24 h at 470 °C	39
BAS	1 h at 580 °C	81
BBS	24 h at 390 °C	59
BS	24 h at 450 °C	42

## Data Availability

The original contributions presented in the study are included in the article, further inquiries can be directed to the corresponding authors.

## Abbreviations

The following abbreviations are used in this manuscript:

DTA	Differential Thermal Analysis
XRD	X-ray Diffractometry
ICDD	International Centre for Diffraction Data
ICSD	Inorganic Crystal Structure Database
